# Effect of Traditional Chinese Medicine on Long-Term Outcomes of Snakebite in Taiwan

**DOI:** 10.3390/toxins12020132

**Published:** 2020-02-20

**Authors:** Teng-I Huang, Ching-Liang Hsieh

**Affiliations:** 1 Department of Chinese Medicine, China Medical University Hospital, Taichung 40447, Taiwan; cocoisme1@gmail.com; 2 Chinese Medicine Research Center, China Medical University, Taichung 40402, Taiwan; 3 Graduate Institute of Acupuncture Science, College of Chinese Medicine, China Medical University, Taichung 40402, Taiwan

**Keywords:** traditional Chinese medicine, long-term effects, venom, snakebites

## Abstract

Herein, we review the characteristics of the six predominant venomous snakes in Taiwan and the effects of traditional Chinese medicine on the long-term outcomes of snakebite venom. We electronically searched databases, including PubMed, ClinicalKey, China National Knowledge Infrastructure, National Digital Library of Theses and Dissertations in Taiwan, and Airiti Library, from their inception to November 2019 by using the following Medical Subject Headings’ keywords: snakebite, long-term, chronic, Chinese medicine, CAM, herb, and Taiwan. The most common long-term effects of snakebite envenomation include “migraine-like syndrome”, brain injuries caused by hypoxia or intracranial hemorrhage, and chronic kidney disease. In addition, hypopituitarism is also worth mentioning. Traditional Chinese medicine can potentially be used in a complementary or alternative treatment for these effects, but additional studies are needed.

## 1. Introduction

Snakebite envenomation is a rare medical issue, with an incidence rate lower than that of other diseases. However, it can lead to serious complications or death. In 2009, the World Health Organization included snakebites in its list of “neglected tropical diseases” [1,2]. Annually, 1.8–2.7 million cases of snakebite envenomation occur and 94,000 people are killed worldwide. The incidence of snakebite envenomation is particularly high in South and South-East Asia [3].

Taiwan is located in a subtropical region near South-East Asia. The climate in Taiwan is warm and humid and the topography is diverse, which is suitable for the survival of various snake species [4]. In Taiwan, more than 20 snake species are venomous, six of which are particularly prevalent and can be classified into two major venomous snake families: *Bungarus multicinctus* and *Naja atra*, which are elapids; and *Trimeresurus stejnegeri*, *Deinagkistrodon acutus*, *T. mucrosquamatus*, and *Daboia siamensis*, which are vipers [5].

*T. stejnegeri* (red tail Bamboo Viper) is widely distributed and is the only major venomous snake in Taiwan that is not a protected species [5]. It is arboreal and usually active at low altitudes in mountains, bamboo groves, or orchards [6]. *De. acutus* (Hundred-Pace snake) is mostly distributed in Eastern and Southern Taiwan and primarily inhabits medium altitude mountains and forests [7,8]. *T. mucrosquamatus* (Taiwan Habu) is widely distributed in low-to-medium altitude areas in Taiwan [9]. It is nocturnal and highly aggressive, and inhabits cool places such as caves, farmlands, bushes, and riversides [4]. *B. multicinctus* (Taiwanese Krait [10]) is distributed in low altitude areas in Taiwan. This nocturnal snake inhabits humid environments, such as marshy areas or the humid areas of fields [4]. *N. atra* (Chinese cobra [11]) is distributed in low altitude areas in Taiwan. It is nocturnal and primarily active in bushes or agricultural fields and characterized by a flattened and raised body when angry or being frightened. *Da. siamensis* (Russell’s Viper) is relatively rare and mostly distributed in Southern Taiwan. In contrast to *B. multicinctus*, this nocturnal snake inhabits dry environments such as open fields, grasslands, or drylands [8]. To see the pictures of the six predominant venomous snake species in Taiwan, please check the link below [12].

According to the pathological effects of snake venoms and the clinical manifestations, these six species can be divided into three subgroups [13]: hemotoxic, neurotoxic, and mixed types. *T. stejnegeri*, *De. acutus*, and *T. mucrosquamatus* belong to the hemotoxic subgroup. The venoms of *T. stejnegeri* and *T. mucrosquamatus* are similar in that they are mainly composed of phospholipase A2 (PLA2s), fibrinogenases, and platelet aggregation inhibitors. However, *T. mucrosquamatus* venom exhibits higher toxicity than *T. stejnegeri* venom [5]. The venom of *De. acutus* is mainly composed of snake venom metalloproteinase (SVMPs), C-type lectins, PLA2s, and snake venom serine protease (SVSPs). The hemotoxic-type venoms exhibit both anticoagulant and coagulant effects, which would induce ischemic or hemorrhagic symptoms [13,14]. *B. multicinctus* and *N. atra* venoms are neurotoxic. The venom of *B. multicinctus* is mainly composed of α-bungarotoxin and β-bungarotoxin [5,15], while the venom of *N. atra* is mainly composed of PLA2s, postsynaptic neurotoxins, and cardiotoxins [16,17]. The neurotoxic-type venoms affect neuropeptide secretion and can induce neurological symptoms such as ptosis, dysarthria, dysphagia, paraplegia, respiratory failure, or shock [18,19]. Furthermore, direct contact of the neurotoxins with the eyes may cause corneal ulcers, ophthalmia, or blindness [17]. *Da. siamensis* venom is mainly composed of SVMPs, snake venom hyaluronidases (SVHYs), PLA2, factor V, and X activators [5,20]. It demonstrates both hemotoxic and neurotoxic characteristics [21,22].

Currently, the most effective treatment for snakebite envenomation is injection with the appropriate antivenoms [23]. In Taiwan, four types of antivenom are available: a bivalent antivenom against *T. stejnegeri* and *T. mucrosquamatus*, a bivalent antivenom against *N. atra* and *B. multicinctus*, an antivenom against *De. acutus*, and an antivenom against *Da. Siamensis* [23,24]. In most cases, acute symptoms induced by snakebite envenomation can be relieved within days after antivenom injection. However, sequelae or delayed pathological effects could last for months or years [11,25,26,27,28,29].

Taiwan has a large venomous snake population, and research on the treatment of long-term effects after snakebite envenomation has been limited. Thus, we summarize the clinical manifestations and the effects of traditional Chinese medicine on the long-term outcomes of snakebite envenomation in Taiwan.

## 2. Results and Discussion

### 2.1. Clinical Manifestations of Snake Envenomation in Taiwan

Despite the fact that the six predominant venomous snake species in Taiwan can be divided into hemotoxic, neurotoxic, and mixed-type subgroups, symptoms following snakebite envenomation from different species share much in common. Local effects include local pain, petechiae, ecchymosis, swelling, blistering, infection (cellulitis or abscess), and cutaneous or muscle necrosis. Local effects induced by envenomation of *De. acutus*, *T. mucrosquamatus*, and *N. atra* are especially severe and may progress to compartment syndrome [5,16,30]. A report described the case of a 41-year-old woman who developed necrotizing fasciitis following *De. acutus* envenomation in Taiwan [14]. A 60-year-old man was reported to develop respiratory failure after a *T. mucrosquamatus* bite; the bite site, on the occipital lobe, demonstrated severe edema, which was followed by acute airway obstruction and respiratory failure [31].

Systemic effects include hypotension and hemostatic alterations that may result in ischemic stroke, intracranial hemorrhage, or disseminated intravascular coagulation (DIC).

Other symptoms highly associated with envenomation by particular species include acute kidney injury and acute pituitary failure, which are the most relevant to *Da. siamensis* envenomation [32,33]; ptosis and respiratory failure, which are the most relevant to *B. multicinctus* envenomation; and ophthalmia, which is the most relevant to *N. atra* envenomation. The main toxins and clinical manifestations of snake envenomation in Taiwan are summarized in Table 1.

### 2.2. Long-Term Effects of Snake Envenomations

#### 2.2.1. Migraine-Like Syndrome

Migraine-like syndrome following snakebite envenomation was reported in a study from Sri Lanka. It revealed that 112 of 816 snakebite victims presented with long-term complications, and 46 patients (5.6%) developed “migraine-like-syndrome”, which presented as headaches, dizziness, and sensitivity to light [36]. Hypercoagulability is suspected to be related to migraine pathophysiology, particularly migraine aura [35]. The pathogenesis of snake venom-induced migraine-like syndrome remains unclear. It has been hypothesized that it is caused by hemotoxins, which have procoagulant properties and may affect cranial blood vessels. *T. stejnegeri*, *T. mucrosquamatus*, and *Da. siamensis* venoms contain an abundance of procoagulant proteins, such as Factor V activator, Factor IX activator, Factor X activator, thrombin-like enzymes, and prothrombin activator [20,37,39].

In traditional Chinese medicine, the pathogenic factor of migraine is considered to be blood stasis, which could correspond to hypercoagulability. Therefore, to treat migraines, traditional Chinese medicine practitioners prescribe herbs, such as *Angelica sinensis* (Dang gui) and *Ligusticum chuanxiong* (Chuan xiong), that promote blood flow and dispel blood stasis [40]. The pharmacological activities of *A. sinensis* and *L. chuanxiong* include antioxidant, anti-inflammatory and antinociceptive effects [41,42]. Ligustilide, one of the main active components of *A. sinensis* and *L. chuanxiong*, is capable of regulating the release of calcitonin gene-related protein (CGRP): a highly potent vasoactive peptide that can cause vasodilatation [42]. In addition, *Xiang Fu Chuan Xiong Cha*, an ancient formula, composed of *Cyperi Rhizoma* and *Chuanxiong Rhizoma* at a 2:1 weight ratio, was used for curing migraines and headaches. A recent animal study demonstrated that *Cyperi Rhizoma* and *Chuanxiong Rhizoma* (CRCR) significantly increased cerebral blood flow and decreased several substances involved in neurogenic inflammation, thereby relieving migraines [43]. *Fructus Viticis* (Man jing zi) is another traditional Chinese medicine that is commonly used for curing migraines and headaches. However, *Fructus Viticis* does not increase cerebral blood flow but regulates neuropeptides such as 5-hydroxytryptamine (5-HT), γ-aminobutyric acid (GABA), calcitonin gene-related peptide, and substance P [44].

#### 2.2.2. Brain Injuries

Snakebite envenomation may lead to serious neurological complications, including acute respiratory failure (due to muscle paralysis) [19], ischemic stroke [45], and intracranial hemorrhage [46], which can result in brain injuries.

• Respiratory failure

Respiratory failure is caused by neurotoxic envenomation in most cases. Neurotoxins are a major component of the venoms of most elapid snakes such as *B. multicinctus* and *N. atra*. These toxins can be divided into two groups: α-neurotoxins and β-neurotoxins. α-neurotoxins act on postsynaptic terminals, and belong to the family of three-finger toxins that block acetylcholine receptors; β-neurotoxins act on presynaptic terminals, and belong to the family of heterodimeric PLA2s that inhibit the release of acetylcholine from presynaptic terminals [15,38,47]. The combined effects of α-neurotoxins and β-neurotoxins block neuromuscular transmission and result in muscle paralysis. In respiratory muscles, they induce acute respiratory failure [31,48] and result in hypoxic brain injuries.

• Cerebral infarction and intracranial hemorrhage

Hemotoxins are responsible for cerebral infarction and intracranial hemorrhage following snakebite envenomation. Hemotoxins are major components of Viperidae venoms, such as from *T. stejnegeri*, *T. mucrosquamatus*, *De. acutus*, and *Da. siamensis*. These hemotoxins can be categorized into two groups: hemorrhagic or nonhemorrhagic.

Hemorrhagic toxins include SVMPs, SVSPs, PLA2, C-type lectin-like proteins, and α-fibrinogenase, which cause hemorrhages as they have anticoagulant properties, inhibit platelet aggregation, and degrade vessel walls [13,37,47]. A case report described a 22-year-old man who developed intracranial hemorrhage caused by a viper snakebite [48].

Nonhemorrhagic toxins include Factor V activator, Factor IX activator, Factor X activator, prothrombin activator, thrombin-like enzymes, and aggregoserpentin. These toxins cause thrombosis as a result of their procoagulant properties and activation of platelet aggregation [13,37,47]. A case report described a 49-year-old woman who developed acute cerebral infarction following a *T. stejnegeri* snakebite in Taiwan [45]. Both cerebral infarction and intracranial hemorrhage can result in brain injuries that cause long-term sequelae such as hemiplegia or cerebellar ataxia [36,49].

Certain herbs used in traditional Chinese medicine have demonstrated neuroprotective effects and neurogenesis functions [50,51]. In animal studies of immature cortices, *A. sinensis* demonstrated neuroprotective effects following hypoxic brain damage [52]. *Gastrodia elata* can reduce brain edema and neuronal loss, and improve neural stem cell proliferation [53]. Paeoniflorin, extracted from *Paeonia lactiflora*, can suppress neuronal apoptosis and promote neurogenesis [51]. A self-designed formula called Post-Stroke Rehabilitation (PSR)—composed of *Astragalus membranaceus*, *Salvia miltiorrhiza*, *P. lactiflora*, *Cassia obtusifolia*, *L. chuanxiong*, *A. sinensis*, and *Glycyrrhiza uralensis*—demonstrated neuroprotective effects by protecting cultured neurons against N-methyl-D-aspartate excitotoxicity and reducing ischemic injury [54].

#### 2.2.3. Chronic Kidney Disease

Among the six predominant venomous snakes in Taiwan, *Da. siamensis* envenomation is the most relevant to kidney injury. *Da. siamensis* venom primarily demonstrates hemotoxic effects with some neurotoxic effects [5]. The causes of kidney injury are multifactorial and include bleeding, hypotension, intravascular hemolysis, disseminated intravascular coagulation, and nephrotoxicity [5,55].

In most cases, acute kidney injuries caused by snakebite envenomation are healed within days or months after treatment [55]. However, in some cases, acute kidney injuries develop into chronic renal disease. A descriptive study in Sri Lanka revealed that 20 (37%) of 54 patients who had acute kidney injury after a snakebite developed chronic renal disease. Furthermore, the renal histology of six patients demonstrated predominant glomerular sclerosis and interstitial nephritis [56].

A large retrospective study in Taiwan reported that traditional Chinese medicine reduced end-stage renal disease risk by 60% in patients with chronic kidney disease [57]. Some herbs such as *As. membranaceus*, *Ophiocordyceps sinensis*, *Rheum palmatum*, and Cortex Moutan (root bark of *P. suffruticosa*), have displayed benefits in treating kidney injuries [58]. Furthermore, *As. membranaceus*, with strong anti-inflammatory effects, can reduce proteinuria and attenuate kidney injury in several animal models [59].

#### 2.2.4. Hypopituitarism

Hypopituitarism is not a common complication following snakebite envenomation. Nevertheless, there were several case reports that revealed that victims suffered from hypoglycemia, fatigue, loss of libido (male), and amenorrhea (female) after a Russell’s viper bite [27,60]. A retrospective study in Burma reported that seven of 24 individuals had clinical features of hypopituitarism, while four of 24 individuals had pituitary hormonal abnormalities without evident manifestations [61]. In India, a 45-year-old male was reported to develop delayed hypopituitarism after a Russell’s viper bite. The patient complained about lethargy and loss of appetite for six months, and laboratory data showed a decrease of T4, LH and cortisol. As a result of adrenal and thyroid deficiency, a brain MRI was arranged. The image demonstrated an empty sella with a thinned out pituitary gland [62]. The imaging of pituitary damage caused by snakebite is different from that of postpartum necrosis (Sheehan’s syndrome), which usually showed hemorrhage into a normal size pituitary. Venoms of other snakes reveal similar hemotoxic effects on intravascular coagulation to Russell’s viper venom. However, Russell’s viper envenomation is particularly relevant to pituitary damage [63].

Hypopituitarism following snakebite envenomation is considered to be related to acute kidney injury and chronic kidney disease. A prospective observational study in India showed that nine of 96 patients developed hypopituitarism after a Russell’s viper bite. All of the nine patients suffered from acute kidney injury and had dialysis, while five (55.56%) of the nine developed varying degrees of chronic kidney disease on long-term follow up [64].

Therapeutic agents currently used for hypopituitarism include hypophysis hormones and steroids [62]. In traditional Chinese medicine, the pathogenic factor of hypopituitarism is considered to be ”kidney deficiency”. Hence, to treat hypopituitarism, practitioners prescribe herbs or a formula to nourish both kidney-yang and kidney-yin, such as *Er-Xian Decoction* (EXD). EXD is a traditional Chinese medicine formula composed of *Herba Epimedii* (Yinyanghuo), *Radix morindae Officinalis* (Bajitian), *A. Sinensis*, *R. Anemarrhenae* (Zhimu), *Phellodendri Chinensis Cortex* (Huangbo), and *R. Curculiginis* (Xianmao). It was usually used to treat climacteric syndrome and was proved to be effective to trigger the hypothalamic-pituitary-testicular axis and enhance levels of GnRH and LH in an animal model [65]. A study in China showed that *Tiannianyin* (TNY), a Chinese medicinal formula, can elevate PCNA expression in the anterior pituitary cells of aging rats, and enhance levels of testosterone [66]. These findings are summarized in Table 2.

## 3. Conclusions

Envenomation by the six prevalent venomous snakes in Taiwan can lead to long-term effects such as headaches, dizziness, brain injuries, chronic kidney disease, and hypopituitarism. TCM can relieve the symptoms mentioned above, which is suggested to result from its antioxidative, anti-inflammatory and antinociceptive properties. In addition, since TCM treatments use acupuncture or natural products in general, the cost is lower than of Western medicine.

TCM is symptomatic medicine; its treatment mainly focuses on the symptoms of the diseases. The characterization of TCM is pattern identification and treatment, which is different from disease identification and treatment in the Western medicine, which emphasizes the etiology of the disease. Therefore, it is suggested that in the acute stage of snakebite envenomation, Western medicine such as antivenoms and supportive treatment must be used. In addressing the long-term effects of snakebite envenomation, such as “migraine-like syndrome”, TCM could be a complementary treatment. Therefore, how to combine Western medicine and TCM to produce a more beneficial treatment for the patients with snakebite envenomation could be an issue in the future.

## 4. Materials and Methods

**Figure 1:**
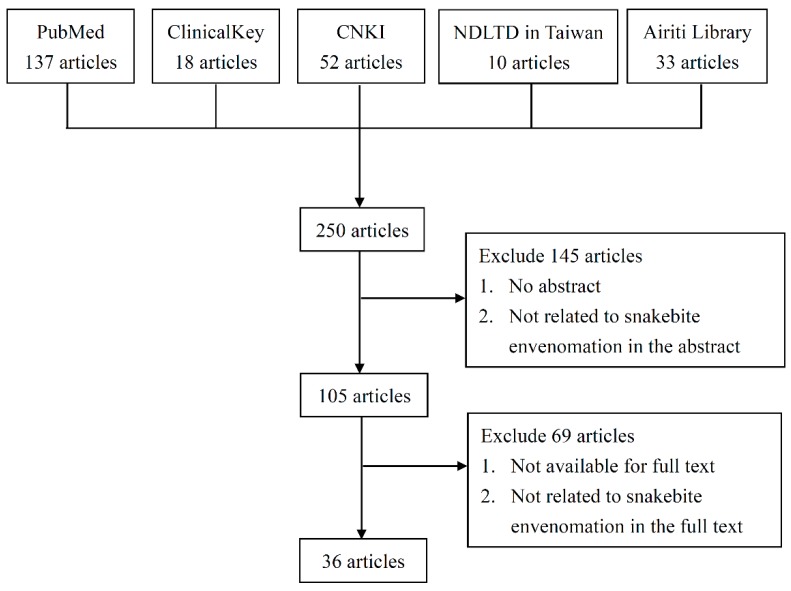
Flow chart of the search processes. CNKI: China National Knowledge Infrastructure; NDLTD: National Digital Library of Theses and Dissertations in Taiwan.

We searched the PubMed, ClinicalKey, China National Knowledge Infrastructure, National Digital Library of Theses and Dissertations in Taiwan, and Airiti Library database from their inception to November 2019. Medical Subject Headings’ keywords included “snakebite”, “long-term”, “chronic”, “Chinese medicine”, “CAM”, “herb”, and “Taiwan”. Languages were limited to English and Chinese. The filter process was first carried through the website search engine, which yielded 250 articles. We manually screened the studies and excluded 145 articles either for lack of abstract, or the abstract not being related to snakebite envenomation. Another 69 articles were excluded because the full text was not available or it was not related to snakebite envenomation. In total, 36 articles were included in the study, including prospective and retrospective studies related to snakebite envenomation. A flowchart of the search process is displayed in Figure 1. For detailed information of the articles we yielded and excluded, please check the Supplementary Materials Table S1. Regarding the images of the six predominant venomous snakes in Taiwan, the information source for this study has been shown in reference [12].

## Figures and Tables

**Table 1:** The main toxins and clinical manifestations of snakebite envenomation in Taiwan.

**Hemotoxic**				
**Snake Species**	**Main Toxins**	**Antivenom**	**Major Clinical Manifestations**	**References**
*T. stejnegeri* *T. mucrosquamatus*	PLA2s, fibrinogenases, and platelet aggregation inhibitors	A	Local effects (local pain, petechiae, ecchymosis, swelling, blistering, necrosis). Systemic effects (hypotension, ischemic stroke, intracranial hemorrhage or disseminated intravascular coagulation).	[13,34,35,36,37]
*De. acutus*	SVMPs, C-type lectins, PLA2s and SVSPs	C		
**Neurotoxic**				
**Snake Species**	**Main toxins**	**Antivenom**	**Major Clinical Manifestations**	**References**
*B. multicinctus*	α-bungarotoxin and β-bungarotoxin	B	Neurological symptoms (ptosis, dysarthria, dysphagia, blurred vision). Respiratory failure.	[5,15,38]
*N. atra*	PLA2s, postsynaptic neurotoxins and cardiotoxins	B	Severe local tissue swelling and necrosis. Ophthalmia.	[16,17]
**Mixed (Hemotoxic and Neurotoxic)**				
**Snake Species**	**Main toxins**	**Antivenom**	**Major Clinical Manifestations**	**References**
*Da. siamensis*	SVMPs, SVHYs, PLA2, factor V, and X activators	D	Hemotoxic effects (pain, swelling, intravascular hemolysis, hypotension). Neurological symptoms. Others (AKI, hypopituitarism).	[5,20,33]

A: a bivalent antivenom against T. stejnegeri and T. mucrosquamatus, B: a bivalent antivenom against N. atra and B. multicinctus; C: an antivenom against De. acutus; D: an antivenom against Da. siamensis. Abbreviations: T. stejnegeri: Trimeresurus stejnegeri; T. mucrosquamatus: Trimeresurus mucrosquamatus; De. acutus: Deinagkistrodon acutus; B. multicinctus: Bungarus multicinctus; N. atra: Naja atra; Da. siamensis: Daboia siamensis; PLA2s: phospholipase A2; SVMPs: snake venom metalloproteinase; SVSPs: snake venom serine protease; SVHYs: snake venom hyaluronidases; AKI: acute kidney injury.

**Table 2:** Effects of traditional Chinese medicine (TCM) on long-term outcomes of snake envenomation.

Long-Term Sequelae	Potential TCM Treatment	Possible Mechanism	References
“Migraine-like syndrome”	*A. sinensis, L. chuanxiong*	Regulate the release of CGRP	[41,42]
	*CRCR*	Increases cerebral blood flow and decreased several substances involved in neurogenic inflammation	[43]
	*F. Viticis*	Regulates neuropeptides	[44]
Brain injuries secondary to hypoxia, ischemic stroke or ICH	*A. sinensis*	Neuroprotective effects	[52]
	*G. elata*	Reduce brain edema and neuronal loss and improve neural stem cell proliferation	[53]
	*P. lactiflora*	Suppress neuronal apoptosis and promote neurogenesis	[51]
	*PSR*	Neuroprotective effects	[54]
CKD	*As. Membranaceus* *O. sinensis* *R. palmatum* *Cortex Moutan*	Unclear	[58]
Hypopituitarism	*EXD*	Trigger hypothalamic–pituitary–testicular axis	[65]
	*TNY*	Elevate PCNA expression	[66]

Abbreviations: TCM: Traditional Chinese medicine; ICH: Intracranial hemorrhage; CKD: Chronic Kidney Disease; A. sinensis: Angelica sinensis; L. chuanxiong: Ligusticum chuanxiong; CRCR: Cyperi Rhizoma and Chuanxiong Rhizoma; F. Viticis: Fructus Viticis; G. elata: Gastrodia elata; P. lactiflora: Paeonia lactiflora; PSR: Post-Stroke Rehabilitation; As. membranaceus: Astragalus membranaceus; O. sinensis: Ophiocordyceps sinensis; R. palmatum: Rheum palmatum; EXD: Er-Xian Decoction; TNY: Tiannianyin; CGRP: calcitonin gene-related protein; PCNA: Proliferating cell nuclear antigen.

## Supplementary Materials

The following are available online at https://www.mdpi.com/2072-6651/12/2/132/s1, Table S1: Review Matrix on the Use of Effect of traditional Chinese medicine on long-term outcomes of snakebite in Taiwan.
