# Supplementary material for: Effect of Traditional Chinese Medicine on Long-Term Outcomes of Snakebite in Taiwan
Source: Toxins (Basel). 2020 Feb 20;12(2):132. doi: 10.3390/toxins12020132 (PMC7076781; doi:10.3390/toxins12020132)
Supplement: Supplementary file 1 [file toxins-12-00132-s001.pdf]

Supplementary Materials: Effect of Traditional Chinese Medicine on Long-Term Outcomes of Snakebite in Taiwan

Teng-I Huang and Ching-Liang Hsieh *

Table S1. Review Matrix on the Use of Effect of traditional Chinese medicine on long-term outcomes of snakebite in Taiwan.

Reference				Filter	Excluded Reason
Author(s)	Title	Journal/Year Published	Database		
Zhang H, Li C, Kwok ST, Zhang QW, Chan SW	A Review of the Pharmacological Effects of the Dried Root of Polygonum cuspidatum (Hu Zhang) and Its Constituents.	Evid Based Complement Alternat Med. 2013	PubMed		Not related to snakebite envenomation in the abstract
Fu R, Zhang Y, Peng T, Guo Y, Chen F.	Phenolic composition and effects on allergic contact dermatitis of phenolic extracts Sapium sebiferum (L.) Roxb. leaves.	J Ethnopharmacol. 2015	PubMed		Not related to snakebite envenomation in the abstract
Fu R, Zhang YT, Guo YR, Huang QL, Peng T, Xu Y, Tang L, Chen F.	Antioxidant and anti-inflammatory activities of the phenolic extracts of Sapium sebiferum (L.) Roxb. leaves.	J Ethnopharmacol. 2013	PubMed		Not related to snakebite envenomation in the abstract
Ni YS, Sun AZ, Bao JY.	[503 cases of pallas pit viper bite with traditional Chinese medicine and Western medicine treatment].	Zhong Xi Yi Jie He Za Zhi. 1990	PubMed	Not available for full text	
Miao YN, Chen MC, Huang Z.	[Clinical observation on treatment of snake bite induced disseminated intravascular coagulation by qinwen baidu decoction].	Zhongguo Zhong Xi Yi Jie He Za Zhi. 2003	PubMed	Not available for full text	
Gomes A, Das R, Sarkhel S, Mishra R, Mukherjee S, Bhattacharya S, Gomes A.	Herbs and herbal constituents active against snake bite.	Indian J Exp Biol. 2010	PubMed		Herbs that are not available in Taiwan

Upasani MS, Upasani SV, Beldar VG, Beldar CG, Gujarathi PP.	Infrequent use of medicinal plants from India in snakebite treatment.	Integr Med Res. 2018	PubMed		Herbs that are not available in Taiwan
Yirgu A, Chippaux JP.	Ethnomedicinal plants used for snakebite treatments in Ethiopia: a comprehensive overview.	J Venom Anim Toxins Incl Trop Dis. 2019	PubMed		Herbs that are not available in Taiwan
Habib AG.	Tetanus complicating snakebite in northern Nigeria: clinical presentation and public health implications.	Acta Trop. 2003	PubMed	Not available for full text	
Salama R, Sattayasai J, Gande AK, Sattayasai N, Davis M, Lattmann E.	Identification and evaluation of agents isolated from traditionally used herbs against Ophiophagus hannah venom.	Drug Discov Ther. 2012	PubMed		Herbs that are not available in Taiwan
Coe FG, Anderson GJ.	Snakebite ethnopharmacopoeia of eastern Nicaragua.	J Ethnopharmacol. 2005	PubMed		Herbs that are not available in Taiwan
Vásquez J, Jiménez SL, Gómez IC, Rey JP, Henao AM, Marín DM, Romero JO, Alarcón JC.	Snakebites and ethnobotany in the Eastern region of Antioquia, Colombia--the traditional use of plants.	J Ethnopharmacol. 2013	PubMed		Herbs that are not available in Taiwan
Gómez-Betancur I, Benjumea D.	Traditional use of the genus Renealmia and Renealmia alpinia (Rottb.) Maas (Zingiberaceae)-a review in the treatment of snakebites.	Asian Pac J Trop Med. 2014	PubMed		Herbs that are not available in Taiwan
Habib AG, Gebi UI, Onyemelukwe GC.	Snake bite in Nigeria.	Afr J Med Med Sci. 2001	PubMed	Not available for full text	
Caro D, Ocampo Y, Castro J, Barrios L, Salas R, Franco LA.	Protective effect of Dracontium dubium against Bothrops asper venom.	Biomed Pharmacother. 2017	PubMed		Herbs that are not available in Taiwan
Verdan MH, Stefanello MÉ.	Secondary metabolites and biological properties of Gesneriaceae species.	Chem Biodivers. 2012	PubMed		Not related to snakebite envenomation in the abstract

Wei NN, Lv HN, Wu Y, Yang SL, Sun XY, Lai R, Jiang Y, Wang K.	Selective Activation of Nociceptor TRPV1 Channel and Reversal of Inflammatory Pain in Mice by a Novel Coumarin Derivative Muralatin L from Murraya alata.	J Biol Chem. 2016	PubMed		Not related to snakebite envenomation in the abstract
Gakuya DW, Itonga SM, Mbaria JM, Muthee JK, Musau JK.	Ethnobotanical survey of biopesticides and other medicinal plants traditionally used in Meru central district of Kenya.	J Ethnopharmacol. 2013	PubMed		Herbs that are not available in Taiwan
Fatima S, Javed T, Khalid S, Shaheen N, Aslam N, Latif M, Siddique FA, Bibi S, Mohsin S, Yameen MA, Ali Abid SM, Khan SA, Najam A, Afzal K, Hasan SMF, McCleary RJ, Bin Asad MHH.	Evaluation of different Pakistani medicinal plants for inhibitory potential against Echis carinatus induced Phospholipase A2 toxicity.	Pak J Pharm Sci. 2019	PubMed		Herbs that are not available in Taiwan
Zhu J, Lin S.	[Investigation on folk medicine and health care of She ethnic group in Zhejiang province].	Zhonghua Yi Shi Za Zhi. 2002	PubMed	Not available for full text	
Alam MI, Gomes A.	Adjuvant effects and antiserum action potentiation by a (herbal) compound 2-hydroxy-4-methoxy benzoic acid isolated from the root extract of the Indian medicinal plant ‘sarsaparilla’ (Hemidesmus indicus R. Br.).	Toxicon. 1998	PubMed		Herbs that are not available in Taiwan
Yang LC, Wang F, Liu M.	A study of an endothelin antagonist from a Chinese anti-snake venom medicinal herb.	J Cardiovasc Pharmacol. 1998	PubMed	Not available for full text	
Gori L, Gallo E, Mascherini V, Mugelli A, Vannacci A, Firenzuoli F.	Can estragole in fennel seed decoctions really be considered a danger for human health? A fennel safety update.	Evid Based Complement Alternat Med. 2012	PubMed		Not related to snakebite envenomation in the abstract
Harsha VH, Hebbar SS, Hegde GR, Shripathi V.	Ethnomedical knowledge of plants used by Kunabi Tribe of Karnataka in India.	Fitoterapia. 2002	PubMed		Herbs that are not available in Taiwan

Habib AG, Brown NI.	The snakebite problem and antivenom crisis from a health-economic perspective.	Toxicon. 2018	PubMed	Not related to longterm effects of snakebite envenomation in the abstract
Hornbeak KB, Auerbach PS.	Marine Envenomation.	Emerg Med Clin North Am. 2017	PubMed	Not related to the common snakebite envenomation in Taiwan
Vaiyapuri S, Vaiyapuri R, Ashokan R, Ramasamy K, Nattamaisundar K, Jeyaraj A, Chandran V, Gajjeraman P, Baksh MF, Gibbins JM, Hutchinson EG.	Snakebite and its socio-economic impact on the rural population of Tamil Nadu, India.	PLoS One. 2013	PubMed	No related to TCM
Slagboom J, Kool J, Harrison RA, Casewell NR.	Haemotoxic snake venoms: their functional activity, impact on snakebite victims and pharmaceutical promise.	Br J Haematol. 2017	PubMed	Not related to longterm effects of snakebite envenomation in the full text
Waiddyanatha S, Silva A, Siribaddana S, Isbister GK.	Long-term Effects of Snake Envenoming.	Toxins (Basel). 2019	PubMed	
Jayawardana S, Gnanathasan A, Arambepola C, Chang T.	Chronic Musculoskeletal Disabilities following Snake Envenoming in Sri Lanka: A Population-Based Study.	PLoS Negl Trop Dis. 2016	PubMed	
Williams HF, Layfield HJ, Vallance T, Patel K, Bicknell AB, Trim SA, Vaiyapuri S.	The Urgent Need to Develop Novel Strategies for the Diagnosis and Treatment of Snakebites.	Toxins (Basel). 2019	PubMed	Not related to longterm effects of snakebite envenomation in the full text
Naqvi R.	Snake-bite-induced Acute Kidney Injury.	J Coll Physicians Surg Pak. 2016	PubMed	Not related to longterm effects of snakebite

				envenomation in the full text
He X, Wang X, Fang J, Chang Y, Ning N, Guo H, Huang L, Huang X.	The genus Achyranthes: A review on traditional uses, phytochemistry, and pharmacological activities.	J Ethnopharmacol. 2017	PubMed	Not related to snakebite envenomation in the abstract
Jayawardana S, Arambepola C, Chang T, Gnanathasan A.	Long-term health complications following snake envenoming.	J Multidiscip Healthc. 2018	PubMed	
Galizio NDC, Serino-Silva C, Stuginski DR, Abreu PAE, Sant'Anna SS, Grego KF, Tashima AK, Tanaka-Azevedo AM, Morais-Zani K.	Compositional and functional investigation of individual and pooled venoms from long-term captive and recently wild-caught Bothrops jararaca snakes.	J Proteomics. 2018	PubMed	Not related to longterm effects of snakebite envenomation in the abstract
Silva A, Maduwage K, Sedgwick M, Pilapitiya S, Weerawansa P, Dahanayaka NJ, Buckley NA, Siribaddana S, Isbister GK.	Neurotoxicity in Russell's viper (Daboia russelii) envenoming in Sri Lanka: a clinical and neurophysiological study.	Clin Toxicol (Phila). 2016	PubMed	
Spano SJ, Vohra R, Macias F.	Long-term complications of rattlesnake bites: a telephone survey from Central California.	Wilderness Environ Med. 2014	PubMed	
Freiermuth CE, Lavonas EJ, Anderson VE, Kleinschmidt KC, Sharma K, Rapp-Olsson M, Gerardo C; Copperhead Recovery Workgroup.	Antivenom Treatment Is Associated with Fewer Patients using Opioids after Copperhead Envenomation.	West J Emerg Med. 2019	PubMed	Not related to longterm effects of snakebite envenomation in the abstract
Sebastin Santhosh M, Hemshekhar M, Thushara RM, Devaraja S, Kemparaju K, Girish KS.	Vipera russelli venom-induced oxidative stress and hematological alterations: amelioration by crocin a dietary colorant.	Cell Biochem Funct. 2013	PubMed	Not related to longterm effects of snakebite envenomation in the full text
Plowman DM, Reynolds TL, Joyce SM.	Poisonous snakebite in Utah.	West J Med. 1995	PubMed	Not related to longterm effects of snakebite

				envenomation in the abstract
Gilliam LL, Holbrook TC, Ownby CL, McFarlane D, Sleeper MM, Martin S, Levis K, Payton ME.	Cardiotoxicity, inflammation, and immune response after rattlesnake envenomation in the horse.	J Vet Intern Med. 2012	PubMed	Not related to longterm effects of snakebite envenomation in the abstract
Bachsais N, Boussag-Abib L, Laraba-Djebari F.	Safety and efficiency of active immunization with detoxified antigen against scorpion venom: side effect evaluation.	Inflamm Res. 2017	PubMed	Not related to longterm effects of snakebite envenomation in the abstract
Rodríguez-Abarca S, Corrales G, Chacón D, Guevara M, Esquivel C, Arroyo C, Gómez A.	Morphological alterations caused by manual venom extraction on the main venom gland of Bothrops asper and Crotalus simus snakes (Serpentes: Viperidae): Long-term implications for antivenom production.	Toxicon. 2019	PubMed	Not related to longterm effects of snakebite envenomation in the abstract
Herath HM, Wazil AW, Abeysekara DT, Jeewani ND, Weerakoon KG, Ratnatunga NV, Bandara EH, Kularatne SA.	Chronic kidney disease in snake envenomed patients with acute kidney injury in Sri Lanka: a descriptive study.	Postgrad Med J. 2012	PubMed	
Sinha R, Nandi M, Tullus K, Marks SD, Taraphder A.	Ten-year follow-up of children after acute renal failure from a developing country.	Nephrol Dial Transplant. 2009	PubMed	Not related to snakebite envenomation in the abstract
Gwaltney-Brant SM, Rumbeiha WK.	Newer antidotal therapies.	Vet Clin North Am Small Anim Pract. 2002	PubMed	Not related to snakebite envenomation in the abstract
Smyrnioudis ME, O'Rourke DP, Rosenbaum MD, Brewer KL, Meggs WJ.	Long-term efficacy of pressure immobilization bandages in a porcine model of coral snake envenomation.	Am J Emerg Med. 2014	PubMed	Not related to TCM or herbs

Al-Abdulla I, Casewell NR, Landon J.	Long-term physicochemical and immunological stability of a liquid formulated intact ovine immunoglobulin-based antivenom.	Toxicon. 2013	PubMed	Not related to TCM or herbs
White J.	Venomous animals: clinical toxinology.	EXS. 2010	PubMed	Not related to longterm effects of snakebite envenomation in the abstract
Srivastava RN.	Pediatric renal problems in India.	Pediatr Nephrol. 1987	PubMed	Not related to snakebite envenomation in the abstract
Lavonas EJ, Gerardo CJ, O'Malley G, Arnold TC, Bush SP, Banner W Jr, Steffens M, Kerns WP 2nd.	Initial experience with Crotalidae polyvalent immune Fab (ovine) antivenom in the treatment of copperhead snakebite.	Ann Emerg Med. 2004	PubMed	Not related to longterm effects of snakebite envenomation in the abstract
Shaw BA, Hosalkar HS.	Rattlesnake bites in children: antivenin treatment and surgical indications.	J Bone Joint Surg Am. 2002	PubMed	Not related to longterm effects of snakebite envenomation in the abstract
Waikhom R, Sircar D, Patil K, Bennikal M, Gupta SD, Pandey R.	Long-term renal outcome of snake bite and acute kidney injury: a single-center experience.	Ren Fail. 2012	PubMed	
Rahimi-Movaghar V.	Controlled evaluation of injury in an international Safe Community: Kashmar, Iran.	Public Health. 2010	PubMed	Not related to snakebite envenomation in the abstract
Freitas-de-Sousa LA, Amazonas DR, Sousa LF, Sant'Anna SS, Nishiyama MY Jr, Serrano SM,	Comparison of venoms from wild and long-term captive Bothrops atrox snakes	Biochimie. 2015	PubMed	Not related to longterm effects of

Junqueira-de-Azevedo IL, Chalkidis HM, Moura-da-Silva AM, Mourão RH.	and characterization of Batroxrhagin, the predominant class PIII metalloproteinase from the venom of this species.				snakebite envenomation in the abstract
Golay V, Roychowdhary A, Pandey R, Singh A, Pasari A, Abraham A.	Acute interstitial nephritis in patients with viperine snake bite: single center experience of a rare presentation.	Saudi J Kidney Dis Transpl. 2012	PubMed		Not related to longterm effects of snakebite envenomation in the full text
Teixeira PH, Thel Tdo N, Ferreira JM, de Azevedo SM Jr, Junior WR, Lyra-Neves RM.	Local knowledge and exploitation of the avian fauna by a rural community in the semi-arid zone of northeastern Brazil.	J Ethnobiol Ethnomed. 2014	PubMed		Not related to snakebite envenomation in the abstract
Copcu E.	Marjolin's ulcer: a preventable complication of burns?	Plast Reconstr Surg. 2009	PubMed		Not related to snakebite envenomation in the abstract
Bell DJ, Wijegunasinghe D, Samarakoon S, Palipana H, Gunasekera S, de Silva HA, Lalloo DG, Ranawaka UK, de Silva HJ.	Neurophysiological findings in patients 1 year after snake bite induced neurotoxicity in Sri Lanka.	Trans R Soc Trop Med Hyg. 2010	PubMed		
Zanon V, Morri A, Lonati D, Paoli A, Camporesi EM, Bosco G.	HBO_2 in snake envenomation (atrox albinus rattlesnake): a case report in a human.	Undersea Hyperb Med. 2016	PubMed		Not related to longterm effects of snakebite envenomation in the abstract
Jeng JC, Fidler PE, Sokolich JC, Jaskille AD, Khan S, White PM, Street JH 3rd, Light TD, Jordan MH.	Seven years' experience with Integra as a reconstructive tool.	J Burn Care Res. 2007	PubMed		Not related to snakebite envenomation in the abstract
Cowin DJ, Wright T, Cowin JA.	Long-term complications of snake bites to the upper extremity.	J South Orthop Assoc. 1998	PubMed	Not available for full text	

Proby C, Tha-Aung, Thet-Win, Hla-Mon, Burrin JM, Joplin GF.	Immediate and long-term effects on hormone levels following bites by the Burmese Russell's viper.	Q J Med. 1990	PubMed	Not available for full text	
Weber RA, White RR 4th.	Crotalidae envenomation in children.	Ann Plast Surg. 1993	PubMed	Not available for full text	
Carneiro SM, Zablith MB, Kerchove CM, Moura-da-Silva AM, Quissell DO, Markus RP, Yamanouye N.	Venom production in long-term primary culture of secretory cells of the Bothrops jararaca venom gland.	Toxicon. 2006	PubMed		Not related to longterm effects of snakebite envenomation in the abstract
Lewandowski K, Homenda W, Mital A, Complak A, Hellmann A.	Erythrophagocytosis by neutrophils--a rare morphological phenomenon resulting in acquired haemolytic anaemia?	Int J Lab Hematol. 2011	PubMed		Not related to snakebite envenomation in the abstract
Lunyera J, Mohottige D, Von Isenburg M, Jeuland M, Patel UD, Stanifer JW.	CKD of Uncertain Etiology: A Systematic Review.	Clin J Am Soc Nephrol. 2016	PubMed		Not related to snakebite envenomation in the abstract
Naik BN, Bhalla A, Sharma N, Mokta J, Singh S, Gupta P, Rai A, Subbiah S, Bhansali A, Dutta P.	Pituitary dysfunction in survivors of Russell's viper snake bite envenomation: A prospective study.	Neurol India. 2018	PubMed		
Pulimaddi R, Parveda AR, Brahmanpally B, Kalakanda PM, Ramakrishna K, Chinnapaka VRD.	Incidence & prognosis of acute kidney injury in individuals of snakebite in a tertiary care hospital in India.	Indian J Med Res. 2017	PubMed		Not related to longterm effects of snakebite envenomation in the abstract
Tsai YH, Hsu WH, Huang KC, Yu PA, Chen CL, Kuo LT.	Necrotizing fasciitis following venomous snakebites in a tertiary hospital of southwest Taiwan.	Int J Infect Dis. 2017	PubMed		

Bailly C.	Cepharanthine: An update of its mode of action, pharmacological properties and medical applications.	Phytomedicine. 2019	PubMed	Not related to snakebite envenomation in the abstract
Gutiérrez JM, Williams D, Fan HW, Warrell DA.	Snakebite envenoming from a global perspective: Towards an integrated approach.	Toxicon. 2010	PubMed	Not related to longterm effects of snakebite envenomation in the abstract
Weinstein SA, Mirtschin PJ, Tristram H, Lawton L, White J.	Local morbidity from red-bellied black snake (Pseudechis porphyriacus, Elapidae) envenoming: Two cases and a brief review of management.	Toxicon. 2018	PubMed	Not related to the common snakebite envenomation in Taiwan
Mohamed F, Endre ZH, Buckley NA.	Role of biomarkers of nephrotoxic acute kidney injury in deliberate poisoning and envenomation in less developed countries.	Br J Clin Pharmacol. 2015	PubMed	Not related to snakebite envenomation in the abstract
Rajagopala S, Thabah MM, Ariga KK, Gopalakrishnan M.	Acute hypopituitarism complicating Russell's viper envenomation: case series and systematic review.	QJM. 2015	PubMed	Not related to longterm effects of snakebite envenomation in the full text
Tun-Pe, Phillips RE, Warrell DA, Moore RA, Tin-Nu-Swe, Myint-Lwin, Burke CW.	Acute and chronic pituitary failure resembling Sheehan's syndrome following bites by Russell's viper in Burma.	Lancet. 1987	PubMed	
Rathnayaka RMMKN, Ranathunga PEAN, Kularatne SAM.	Kidney injury following envenoming by hump-nosed pit viper (Genus: Hypnale) in Sri Lanka: proven and probable cases.	Trans R Soc Trop Med Hyg. 2019	PubMed	Not related to the common snakebite envenomation in Taiwan
Kaaviya R, Vadivelan M, Balamurugan N, Parameswaran S, Thabah MM.	Community Acquired AKI: A Prospective Observational Study from a Tertiary Level Hospital in Southern India.	Indian J Nephrol. 2019	PubMed	Not related to snakebite

				envenomation in the abstract
Pourreau F, Pinsard M, Goyffon M, Plasse F, Desport E, Thierry A, Touchard G, Bridoux F.	Bilateral renal cortical necrosis with end-stage renal failure following envenoming by Proatheris superciliaris: a case report.	Toxicon. 2014	PubMed	Not related to longterm effects of snakebite envenomation in the abstract
Brown N, Landon J.	Antivenom: the most cost-effective treatment in the world?	Toxicon. 2010	PubMed	Not related to TCM or herbs
Mukhopadhyay S, Ghosh A, Kar M.	Methylglyoxal increase in uremia with special reference to snakebite-mediated acute renal failure.	Clin Chim Acta. 2008	PubMed	Not related to longterm effects of snakebite envenomation in the abstract
Anwar M, Green J, Norris P.	Health-seeking behaviour in Pakistan: a narrative review of the existing literature.	Public Health. 2012	PubMed	Not related to snakebite envenomation in the abstract
Claunch NM, Holding ML, Escallón C, Vernasco B, Moore IT, Taylor EN.	Good vibrations: Assessing the stability of snake venom composition after researcher-induced disturbance in the laboratory.	Toxicon. 2017	PubMed	Not related to snakebite envenomation in the abstract
Fitzgerald KT, Shipley BK, Newquist KL, Vera R, Flood AA.	Additional observations and notes on the natural history of the prairie rattlesnake (Crotalus viridis) in Colorado.	Top Companion Anim Med. 2013	PubMed	Not related to snakebite envenomation in the abstract
Bowie EJ, Owen CA.	The clinical pathology of intravascular coagulation.	Bibl Haematol. 1983	PubMed	Not related to snakebite envenomation in the abstract
Stewart MJ.	South Africa: a toxicologist’s goldmine.	Ther Drug Monit. 2002	PubMed	Not related to snakebite

				envenomation in the abstract
Rogosnitzky M, Danks R.	Therapeutic potential of the biscoclaurine alkaloid, cepharanthine, for a range of clinical conditions.	Pharmacol Rep. 2011	PubMed	Not related to snakebite envenomation in the abstract
Rao KM, Kumar RH, Krishna KS, Bhaskar V, Laxmaiah A.	Diet & nutrition profile of Chenchu population - A vulnerable tribe in Telangana & Andhra Pradesh, India.	Indian J Med Res. 2015	PubMed	Not related to snakebite envenomation in the abstract
Boonpucknavig V, Soontornniyomkij V.	Pathology of renal diseases in the tropics.	Semin Nephrol. 2003	PubMed	Not related to snakebite envenomation in the abstract
Milani Júnior R, Jorge MT, de Campos FP, Martins FP, Bousso A, Cardoso JL, Ribeiro LA, Fan HW, França FO, Sano-Martins IS, Cardoso D, Ide Fernandez C, Fernandes JC, Aldred VL, Sandoval MP, Puorto G, Theakston RD, Warrell DA.	Snake bites by the jararacuçu (Bothrops jararacussu): clinicopathological studies of 29 proven cases in São Paulo State, Brazil.	QJM. 1997	PubMed	Not related to longterm effects of snakebite envenomation in the full text
Silva A, Samarasinghe R, Pilapitiya S, Dahanayake N, Siribaddana S.	Viper bites complicate chronic agrochemical nephropathy in rural Sri Lanka.	J Venom Anim Toxins Incl Trop Dis. 2014	PubMed	
Abib H, Laraba-Djebari F.	Effects of 60Co gamma radiation on toxicity and hemorrhagic, myonecrotic, and edema-forming activities of Cerastes cerastes venom.	Can J Physiol Pharmacol. 2003	PubMed	Not related to longterm effects of snakebite envenomation in the abstract
Sweni S, Meenakshisundaram R, Sakthirajan R, Rajendiran C, Thirumalaikolundusubramanian P.	Acute renal failure in acute poisoning: prospective study from a tertiary care centre of South India.	J Ren Care. 2012	PubMed	Not related to longterm effects of snakebite envenomation in the full text

Dickinson CE, Traub-Dargatz JL, Dargatz DA, Bennett DG, Knight AP.	Rattlesnake venom poisoning in horses: 32 cases (1973-1993).	J Am Vet Med Assoc. 1996	PubMed		Not related to snakebite envenomation in human in the abstract
Bhattacharjee P, Bhattacharyya D.	Characterization of the aqueous extract of the root of Aristolochia indica: evaluation of its traditional use as an antidote for snake bites.	J Ethnopharmacol. 2013	PubMed		Not related to longterm effects of snakebite envenomation in the full text
Golay V, Roychowdhary A, Dasgupta S, Pandey R.	Hypopituitarism in patients with vasculotoxic snake bite envenomation related acute kidney injury: a prospective study on the prevalence and outcomes of this complication.	Pituitary. 2014	PubMed		
Jurkovich GJ, Luterman A, McCullar K, Ramenofsky ML, Curreri PW.	Complications of Crotalidae antivenin therapy.	J Trauma. 1988	PubMed		Not related to longterm effects of snakebite envenomation in the full text
da Silva OA, López M, Godoy P.	Intensive care unit treatment of acute renal failure following snake bite.	Am J Trop Med Hyg. 1979	PubMed		Not related to longterm effects of snakebite envenomation in the full text
Caiaffa WT, Vlahov D, Antunes CM, de Oliveira HR, Diniz CR.	Snake bite and antivenom complications in Belo Horizonte, Brazil.	Trans R Soc Trop Med Hyg. 1994	PubMed		Not related to TCM
da Silva OA, López M, Godoy P.	Bilateral cortical necrosis and calcification of the kidneys following snakebite: a case report.	Clin Nephrol. 1979	PubMed	Not available for full text	

Al-Homrany M.	Epidemiology of acute renal failure in hospitalized patients: experience from southern Saudi Arabia.	East Mediterr Health J. 2003	PubMed		Not related to longterm effects of snakebite envenomation in the full text
Herath N, Wazil A, Kularatne S, Ratnatunga N, Weerakoon K, Badurdeen S, Rajakrishna P, Nanayakkara N, Dharmagunawardane D.	Thrombotic microangiopathy and acute kidney injury in hump-nosed viper (Hypnale species) envenoming: a descriptive study in Sri Lanka.	Toxicon. 2012	PubMed		Not related to longterm effects of snakebite envenomation in the full text
Johnson HA.	The foot that stalled a thousand ships: a controversial case from the 13th century BCE.	J R Soc Med. 2003	PubMed		Not related to snakebite envenomation in the abstract
Boyer LV, Seifert SA, Clark RF, McNally JT, Williams SR, Nordt SP, Walter FG, Dart RC.	Recurrent and persistent coagulopathy following pit viper envenomation.	Arch Intern Med. 1999	PubMed	Not available for full text	
Ilic N, Schmidt BM, Poulev A, Raskin I.	Toxicological evaluation of grains of paradise (Aframomum melegueta) [Roscoe] K. Schum.	J Ethnopharmacol. 2010	PubMed		Not related to snakebite envenomation in the abstract
Denis D, Lamireau T, Llanas B, Bedry R, Fayon M.	Rhabdomyolysis in European viper bite.	Acta Paediatr. 1998	PubMed		Not related to longterm effects of snakebite envenomation in the full text
Makis A, Kattamis A, Grammeniatis V, Sihlimiri P, Kotsonis H, Iliadis A, Siamopoulou A, Chaliasos N.	Pulmonary embolism after snake bite in a child with Diamond-Blackfan anemia.	J Pediatr Hematol Oncol. 2011	PubMed		Not related to longterm effects of snakebite envenomation in the full text

Laothong C, Sitprija V.	Decreased parasympathetic activities in Malayan krait (Bungarus candidus) envenoming.	Toxicon. 2001	PubMed		Not related to longterm effects of snakebite envenomation in the full text
Pyakurel R, Sharma N, Paudel D, Coghill A, Sinden L, Bost L, Larkin M, Burrus CJ, Roy K.	Cause of Death in Women of Reproductive Age in Rural Nepal Obtained Through Community-Based Surveillance: Is Reducing Maternal Mortality the Right Priority for Women's Health Programs?	Health Care Women Int. 2015	PubMed		Not related to snakebite envenomation in the abstract
Harada K, Bando T, Yoshida H, Sato M.	Characteristics of antitumour activity of cepharanthin against a human adenosquamous cell carcinoma cell line.	Oral Oncol. 2001	PubMed		Not related to snakebite envenomation in the abstract
Kaballo BG, Khogali MS, Khalifa EH, Khaiii EA, Ei-Hassan AM, Abu-Aisha H.	Patterns of "severe acute renal failure" in a referral center in Sudan: excluding intensive care and major surgery patients.	Saudi J Kidney Dis Transpl. 2007	PubMed		Not related to snakebite envenomation in the abstract
Nordlander NB.	[How a foot wound could stop the Trojan war].	Lakartidningen. 2004	PubMed	No abstract available	
Mello LF, Barcelos MG, Meohas W, Pinto LW, Melo PA, Nogueira Neto NC, Smith J.	Chronic ulceration of the leg following extensive scarring due to a snake bite complicated by squamous cell carcinoma.	Skeletal Radiol. 2000	PubMed		
Madhusudhanan M, Yusuff AM.	Unusual presentation of chronic idiopathic thrombocytopenic purpura.	Oman Med J. 2008	PubMed		Not related to the common snakebite envenomation in Taiwan
Chowhan JS.	Chronic Snake-Bite.	Ind Med Gaz. 1940	PubMed		Not related to the common snakebite envenomation in Taiwan

Das I.	Chronic Snake-Bite.	Ind Med Gaz. 1939	PubMed		Not related to the common snakebite envenomation in Taiwan
Bartholdi D, Selic C, Meier J, Jung HH.	Viper snakebite causing symptomatic intracerebral haemorrhage.	J Neurol. 2004	PubMed	No abstract available	
Suda M, Yoshikawa Y, Dohi Y.	[Bites, stings and food poisoning].	Nihon Rinsho. 1991	PubMed	No abstract available	
Wolffers I.	Illness behaviour in Sri Lanka: results of a survey in two Sinhalese communities.	Soc Sci Med. 1988	PubMed		Not related to snakebite envenomation in the abstract
Tomov PV.	[Chronic leg ulcer caused by snake bite].	Vestn Khir Im I I Grek. 1989	PubMed	No abstract available	
Anuradhani Kasturiratne, A. Rajitha Wickremasinghe, Nilanthi de Silva, N. Kithsiri Gunawardena, Arunasalam Pathmeswaran, Ranjan Premaratna, Lorenzo Savioli, David G Lalloo, H. Janaka de Silva	The Global Burden of Snakebite: A Literature Analysis and Modelling Based on Regional Estimates of Envenoming and Deaths	PLoS Med. 2008	PubMed		
Hung DZ	Taiwan's venomous snakebite: epidemiological, evolution and geographic differences.	Trans R Soc Trop Med Hyg. 2004	PubMed		
Creer S, Malhotra A, Thorpe RS, Chou WH.	Multiple causation of phylogeographical pattern as revealed by nested clade analysis of the bamboo viper (Trimeresurus stejnegeri) within Taiwan	Mol Ecol. 2001	PubMed		
Chen PC, Huang MN, Chang JF, Liu CC, Chen CK, Hsieh CH	Snake venom proteome and immuno-profiling of the hundred-pace viper, Deinagkistrodon acutus, in Taiwan.	Acta Trop. 2019	PubMed		

Tsai YH, Hsu WH, Huang KC, Yu PA, Chen CL, Kuo LT.	Necrotizing fasciitis following venomous snakebites in a tertiary hospital of southwest Taiwan	Int J Infect Dis. 2017	PubMed
Gutiérrez JM, Calvete JJ, Habib AG, Harrison RA, Williams DJ, Warrell DA.	Snakebite envenoming	Nat Rev Dis Primers. 2017	PubMed
Cheng CL, Mao YC, Liu PY, Chiang LC, Liao SC, Yang CC	Deinagkistrodon acutus envenomation: a report of three cases	J Venom Anim Toxins Incl Trop Dis. 2017	PubMed
Shivaprasad C, Aiswarya Y, Sridevi A, Anupam B, Amit G, Rakesh B, Annie PA, Anish K	Delayed hypopituitarism following Russell's viper envenomation: a case series and literature review	Pituitary. 2019	PubMed
P S Priyamvada, Challa Jaswanth, Bobby Zachariah, Satish Haridasan, Sreejith Parameswaran, Rathinam Palamalai Swaminathan	Prognosis and long-term outcomes of acute kidney injury due to snake envenomation	Clinical Kidney Journal, 2019	PubMed
Hung DZ, Wu ML, Deng JF, Lin-Shiau SY.	Russell's viper snakebite in Taiwan: ifferences from other Asian countries	Toxicon. 2002	PubMed
Zeng X, Hu J, Liang X, Wu Y, Yan M, Zhu M, Fu Y.	Acute cerebral infarction following a Trimeresurus stejnegeri snakebite: A case report.	Medicine (Baltimore). 2019	PubMed
Bartholdi D, Selic C, Meier J, Jung HH.	Viper snakebite causing symptomatic intracerebral haemorrhage	J Neurol. 2004	PubMed
Srinivasan KG, Srividya S, Usha Nandhini KP, Ramprabananth S	Chronic Pituitary Failure Resembling Sheehan's Syndrome Following a Bite of Russell's Viper. A Case Report	Neuroradiol J. 2010	PubMed
Golay V, Roychowdhary A, Dasgupta S, Pandey R.	Hypopituitarism in patients with vasculotoxic snake bite envenomation related acute kidney injury: a prospective study on the prevalence and outcomes of this complication	Pituitary. 2014	PubMed
Antonypillai CN, Wass JA, Warrell DA, Rajaratnam HN.	Hypopituitarism following envenoming by Russell's vipers (Daboia siamensis and D. russelii) resembling Sheehan's syndrome: first case report from Sri Lanka, a review of	QJM. 2011	PubMed

	the literature and recommendations for endocrine management.				
Bartholdi D, Selic C, Meier J, Jung HH.	Viper snakebite causing symptomatic intracerebral haemorrhage.	J Neurol. 2004	PubMed		
Del Brutto OH	Neurological effects of venomous bites and stings: snakes, spiders, and scorpions	Handb Clin Neurol. 2013	PubMed		
Alok Atreya, Tanuj Kanchan	Human–Snake Encounters and Folk Remedies in Nepal	Wilderness & Environmental Medicine, 2018	ClinicalKey		Herbs not available in Taiwan
Gomes A, Das R, Sarkhel S, Mishra R, Mukherjee S, Bhattacharya S, Gomes A	Herbs and herbal constituents active against snake bite.	Indian J. Exp. Biol., 2010	ClinicalKey	Not available for full text	
Salama R, Sattayasai J, Gande AK, Sattayasai N, Davis M, Lattmann E	Identification and evaluation of agents isolated from traditionally used herbs against Ophiophagus hannah venom.	Drug Discov. Ther., 2012	ClinicalKey		Herbs not available in Taiwan
David A. Warrell DM, DSc, FRCP, FRCPE, FMedSci	Venomous Bites, Stings, and Poisoning	Infectious Disease Clinics of North America, 2019	ClinicalKey		
Upasani MS, Upasani SV, Beldar VG, Beldar CG, Gujarathi PP	Infrequent use of medicinal plants from India in snakebite treatment.	Integr. Med. Res., 2018	ClinicalKey		Herbs not available in Taiwan
Butt MA, Ahmad M, Fatima A, Sultana S, Zafar M, Yaseen G, Ashraf MA, Shinwari ZK, Kayani S	Ethnomedicinal uses of plants for the treatment of snake and scorpion bite in Northern Pakistan.	J Ethnopharmacol, 2015	ClinicalKey		Herbs not available in Taiwan
Kadir MF, Karmoker JR, Alam MR, Jahan SR, Mahbub S, Mia MM	Ethnopharmacological survey of medicinal plants used by traditional healers and indigenous people in chittagong hill tracts, bangladesh, for the treatment of snakebite.	Evid Based Complement Alternat Med, 2015	ClinicalKey		Acute stage(Antivenom)
Yirgu A, Chippaux JP	Ethnomedicinal plants used for snakebite treatments in Ethiopia: a comprehensive overview.	J Venom Anim Toxins Incl Trop Dis, 2019	ClinicalKey		Herbs not available in Taiwan
David A Warrell	Snake bite	Lancet, The, 2010	ClinicalKey		

E.J. Lavonas, W.P. Kerns, C.J. Gerardo, W. Richardson, K. Whitlow, D.J. Berkoff	328: Long-Term Limb Function Outcomes Following Copperhead Snakebite	Annals of Emergency Medicine, 2008	ClinicalKey	The effect of antivenom but not TCM
Susanne J. Spano MD, Rais Vohra MD, Fernando Macias MD	Long-Term Complications of Rattlesnake Bites: A Telephone Survey From Central California	Wilderness & Environmental Medicine, 2014	ClinicalKey	
Jayawardana S, Arambepola C, Chang T, Gnanathasan A	Long-term health complications following snake envenoming.	J Multidiscip Healthc, 2018	ClinicalKey	
Richard T. Tovar, Renee M. Petzel Phar	Herbal Toxicity	Disease-a-Month, 2009	ClinicalKey	Not related to snakebite envenomation
Rajendran Silambarasan, Jeyalatchagan Sureshkumar, Jayaraj Krupa, Singamoorthy Amalraj, Muniappan Ayyanar	Traditional herbal medicines practiced by the ethnic people in Sathyamangalam forests of Western Ghats, India	European Journal of Integrative Medicine, 2017	ClinicalKey	Herbs not available in Taiwan
Jeyalatchagan Sureshkumar, Rajendran Silambarasan, Muniappan Ayyanar	An ethnopharmacological analysis of medicinal plants used by the Adiyan community in Wayanad district of Kerala, India	European Journal of Integrative Medicine, 2017	ClinicalKey	Herbs not available in Taiwan
Coe FG, Anderson GJ	Snakebite ethnopharmacopoeia of eastern Nicaragua.	J Ethnopharmacol, 2005	ClinicalKey	Herbs not available in Taiwan
Yi-Chiao Lee, Ting-Hao Wang, Shih-Yu Chen, Hsiang-Ling Lin, Ming-Yen Tsai	Management of viral oral ulcers in children using Chinese herbal medicine: A report of two cases	Complementary Therapies in Medicine, 2017	ClinicalKey	Not related to snakebite envenomation
Marilena Gilca, George Sorin Tiplica, Carmen Maria Salavastru	Traditional and ethnobotanical dermatology practices in Romania and other Eastern European countries	Clinics in Dermatology, 2018	ClinicalKey	Not related to snakebite envenomation
Chi-Wen Juan	Venomous Snake Bites in Taiwan	Journal of Emergency and Critical Care Medicine, 2012	Airiti Library	
Bor-Lin Miao, Ruey-Jen Huang, Men-Shing Hu, Ming-Yi Liau	Venomous Snake Bites in Taiwan (1988-1991) (Chinese)	Chinese Journal of Public Health, 1995	Airiti Library	Not related to longterm effects of snakebite

				envenomation in the abstract
Kao-Ping Chang, Chung-Sheng Lai, Sin-Daw Lin	Management of Poisonous Snake Bites in Southern Taiwan	The Kaohsiung Journal of Medical Sciences, 2007	Airiti Library	
Meng-Si Wu, Jiunn-Tat Lee, Tzong-Bor Sun, Li-Fu Cheng, Chien-Hsing Wang, Hon-Da Hsu, Chieh-Chi Huang, Sou-Hsin Chien	Treatment of Venomous Snakebites in Eastern Taiwan	The Journal of Plastic Surgical Association R.O.C., 2009	Airiti Library	Not related to longterm effects of snakebite envenomation in the abstract
Yu-Chung Shih, Hsu Ma, Fa-Lai Yeh, Jin-The Lin, Chih-Hung Hwang, Mau-Shan Wang, Cherng-Kang Perng, Bing-Hwei Shen, Chien-Hua Chen	Risk Factors of Surgical Intervention in the Management of Venomous Snakebite in Northern Taiwan	The Journal of Plastic Surgical Association R.O.C., 2006	Airiti Library	Not related to longterm effects of snakebite envenomation in the abstract
Chao-Hung Ho	Common snake bite in Taiwan (Chinese)	Health World, 2001	Airiti Library	Not related to longterm effects of snakebite envenomation in the abstract
Ko Cheng	Prevention and treatment of bite by viper snake in Taiwan (Chinese)	Family Medicine & Primary Medical Care, 2018	Airiti Library	Not related to longterm effects of snakebite envenomation in the abstract
Yen-Long Chen	I'm Innocent- Simple Identification of the Similar Species of Poisonous and Nonpoisonous Snakes in Taiwan (Chinese)	Nature Conservation Quarterly, 2011	Airiti Library	Not related to longterm effects of snakebite envenomation in the abstract
Wei-Jone Liang, Yeou-Ming Tung, Hsian-Jenn Wang	Treatment of Snakebite in Taiwan-Analysis of 100 Cases (Chinese)	Journal of Medical Sciences, 1992	Airiti Library	Not related to longterm effects of snakebite

					envenomation in the abstract
Wei-Heng Chang, Francis F.S. Wu, Chi-Wen Juan, Tom C.N. Lee, Chu-Chung Chou, Sai-Kit Ho	Snakebite - Induced Transient Arterial Hypertension	Journal of Emergency and Critical Care Medicine, 2000	Airiti Library		Not related to longterm effects of snakebite envenomation in the abstract
Chuan-Hui Yu, Jer-Kan Wu	Nursing Care of Patients with Poisonous Snakebites (Chinese)	VGH Nursing, 2000	Airiti Library		Nursing
Ting, M.J.; Chu, C.C.; Hsu, Y.L.; Tian, A.C.; Wang, P.H.	The Respiratory Care for Acute Respiratory Failure in the Patient Envenomed by Bungarus Multicinctus (Chinese)	Journal of Respiratory Therapy, 2017	Airiti Library		
Chia-Meng Yu, Wen-Chen Huang, Kwang-Yi Tung, Hung-Tao Hsiao, Shuan-Yuan Ou	Prognostic Factors of Local Necrosis Due to Poisonous Snakebite-A Clinical Review in Mackay Memorial Hospital	The Journal of Plastic Surgical Association R.O.C., 2005	Airiti Library		Not related to longterm effects of snakebite envenomation in the abstract
Gian-Kaw Lee, Ming-Ling Wu, Jou-Fang Deng, Wei-Jen Tsai, How-Chin Liao, Shiumn-Jen Liao	Taiwan Cobrai(Naja naja atra)injury - Cases analysis of Poison Control Center -	Journal of Taiwan Emergency Medicine, 2000	Airiti Library	Not available for full text	
Chin-Yi Chen, Kung-Ta Lee, Tsu-Hwie Liu, Wen-Hsiung Liu	Callus Induction of Podophyllum pleianthum Hance and the Detection of Podophyllotoxin (Chinese)	Taiwan Journal of Agricultural Chemistry and Food Science, 2004	Airiti Library		Not related to snakebite envenomation in the abstract
Hsu, Y.L.; Wu, C.J.; Chou, T.C.; Hsieh, W.C.; Cheng, Y.F.; Chiang, C.J.	Review and Prospect of Antivenom Serum Manufacturing in Taiwan (Chinese)	Taiwan Epidemiology Bulletin, 2013	Airiti Library		Not related to longterm effects of snakebite envenomation in the abstract

Liu, C.H.; Li, C.J.; Li, J.; Hsu, C.L.; Chen, C.H.; Chang, H.C.; Hsieh, W.C.	Application of snake venom protein and anti-venom serum manufacturing technology (Chinese)	Taiwan Epidemiology Bulletin, 2015	Airiti Library	Not related to longterm effects of snakebite envenomation in the abstract
Ruo-Wen Li	An Anthropological Discourse Analysis of ” Vūruns and Kamavanans” in PaiwanLanguage: A Case Study on In Kanori’s Investigation Reports (1900-1910) (Chinese)	Taiwan Journal of Anthropology, 2015	Airiti Library	Not related to snakebite envenomation
Yaw-Wen Guo, Yu-Chi Lin, Tseng-Yuan Chang, Hwan-Wan Liu	Secretion Mechnism of Snake Venom-Speculation on Clonal Theory (Chinese)	Journal of Medical Sciences, 1996	Airiti Library	Not related to longterm effects of snakebite envenomation in the abstract
Chun-Ching Lin, Jer-Min Lin, Jeng-Jer Yang	The Pharmacognostical Studies on Folkmedicine “Thang-Kau-Tin” from Taiwan (Chinese)	Journal of Chinese Medicine, 1995	Airiti Library	Not related to snakebite envenomation in the abstract
Chi-Wen Juan, Francis FS Wu, Wei-Heng Chang, Tom Chao-Nan Lee, Chu-Chung Chou	A Case of Envenomation by Bungarus Multicinctus	Journal of Emergency and Critical Care Medicine, 1999	Airiti Library	
Ting, M.J.; Tian, A.C.; Chu, C.C.; Hsu, Y.L.; Wang, P.H.	The Respiratory Care for Acute Upper Airway Obstruction and Respiratory Failure in the Patient Bitten by Protobothrops Mucrosquamatus (Chinese)	Journal of Respiratory Therapy, 2017	Airiti Library	
Wen-Ying Chao, Yi-Hung Liu, Jung-Der Wang	Trend of Cumulative Mortality Rate and Years of Potential Life Loss (YPLL) of Poisoning during 1974-1990 in Taiwan (Chinese)	Chinese Journal of Public Health, 1993	Airiti Library	Not related to longterm effects of snakebite envenomation in the abstract

Hung-Chun Che, Yung-Hsiung Lai, Juei-Hsiung Tsai	Acute Renal Failure Following Russell's Viper Envenomation: A Report of Two Cases	The Kaohsiung Journal of Medical Sciences, 1988	Airiti Library		
Huei-Lin Huang, Lea-Yea Chuang, Chun-Chang Chang	Immunological Cross-Reactivity of Phospholipase A2 from Snake Venoms	The Kaohsiung Journal of Medical Sciences, 1985	Airiti Library		Not related to longterm effects of snakebite envenomation in the abstract
Yu-Wen Lin, Ming-Yi Lee, Li-Wen Wei, Cheng-Pei Chung	Influence on Chemical Composition of Anoectochilus formosanus Hayata. Water Extract Treated with Multi-Frequency Ultrasound (Chinese)	Journal of China University of Science and Technology, 2019	Airiti Library		Not related to snakebite envenomation in the abstract
Liu, C.K.; Chen, C.P.	Acute renal failure due to snake bite (Chinese)	Kidney and Dialysis, 2004	Airiti Library		No related to TCM
Cheng, S.F.; Lin, H.L.	Epidemiology and treatment of snake bite (Chinese)	Taiwan Medical Journal, 2010	Airiti Library		Not related to longterm effects of snakebite envenomation in the abstract
Yang, H.C.; Li, C.T.	Identification and Quality Control Method of Turtle Shell Flower and Red Tail Snake Venom in Taiwan (Chinese)	Taiwan Epidemiology Bulletin, 2016	Airiti Library		Not related to longterm effects of snakebite envenomation in the abstract
Wen-Loung Lin, Hui-Yun Tseng, Chii-Jenq Tzeng	Fast, Accurate and Safe Measurement of Snake Body Length by Computer Image Analysis	Journal of the Experimental Forest of National Taiwan University, 2006	Airiti Library		Not related to snakebite envenomation in the abstract
Kun-Jong Lee, Gieh-Hwa Lu	From "The Serpent's Lair" to "The Axis of Evil": Doubts about American Rhetoric in Korean University Classes (Chinese)	CHUNG WAI LITERARY, 2003	Airiti Library		Not related to snakebite envenomation in the abstract

Liu, C.H.; Chiang, T.H.; Lien, W.C.; Liu, T.P.	Analysis of rickets using anti-venom serum in Taiwan from 2002 to 2005 (Chinese)	Taiwan Epidemiology Bulletin, 2009	Airiti Library		Not related to longterm effects of snakebite envenomation in the abstract
Tsung-Hsien Tsai	The Development of Buddhism in Mt. Sheshan from the Fifth to Seventh Centuries (Chinese)	Bulletin of Historical Research, 2016	Airiti Library		Not related to snakebite envenomation in the abstract
Wen-Pei Tseng	Studies on Signal Transduction Pathway of β-Bungarotoxin-induced Neurotoxicity in the Cultured Cerebellar Granule Neurons	Doctoral thesis, National Taiwan University, June 2002	NTLTD		Not related to longterm effects of snakebite envenomation in the full text
Yen-Chia Chen	Study on the Antivenom Treatment and Prognosis of Patients with Crotaline Snake Envenoming in Taiwan	Master thesis, National Yang-Ming University, June 2007	NTLTD	Not available for full text	
Chu, Y.P.	Taiwan Banded Krait β-Bungarotoxins: Novel Isotoxins, Targeting and Gene Organization	Master thesis, National Sun Yat-sen University, May 2002	NTLTD		Not related to longterm effects of snakebite envenomation in the full text
Dong-Zong Hung	Studies on the Diagnosis, Treatment and Toxic Mechanism of Taiwan Venomous Snakebites	Doctoral thesis, National Taiwan University, 2002 July	NTLTD		
Chun-Yu Jen	Role of Taiwan cobra cardiotoxins internalization on cardiomyocyte H92C cells cytotoxicity	Master thesis, National Tsing Hua University, 2008 Aug.	NTLTD		Not related to longterm effects of snakebite envenomation in the full text

Ying-Hui Wang	Taiwan Cobra Venom Induced Intracellular Calcium Increase and Its Biological Implication	Master thesis, National Tsing Hua University, 2003 July	NTLTD		Not related to longterm effects of snakebite envenomation in the full text
-	A Study on the Relationship between Chemical Structure and Lethal Effect of Taiwan Cobra Neurotoxin (Chinese)	-	NTLTD	Not available for full text	
-	Effects of Trimeresurus mucrosquamatus and Deinagkistrodon acutus Venom on Blood Coagulation and Platelet Red Blood Cell Agglutination (Chinese)	-	NTLTD	Not available for full text	
Lin, Y.P.	The cytotoxicity and myotoxicity analysis of toxic prooteins from East and West Taiwan Cobra(Naja naja atra)	Master thesis, National Tsing Hua University, 2004 July	NTLTD		
Lan-Hui Chih	Pharmacological Studies of Phospholipase A2, Isolated from Russell's Vipers, on Smooth Muscle	Master thesis, National Taiwan University, 2001 June	NTLTD		Not related to longterm effects of snakebite envenomation in the full text
Tsao, S.N.; Kung, H.C.	Research progress in treatment of snakebite with traditional Chinese medicine (Chinese)	Journal of Snake, 2019	CNKI		Not related to longterm effects of snakebite envenomation in the full text
Hsu, L.H.; Chu, W.F.; Shen, A.; Chen, L.H.; Chin, C.	Research Progress on Traditional Chinese Medicine External Treatment of the Swelling Caused by Venomous Snakebite (Chinese)	Journal of Jiangxi University of Traditional Chinese Medicine, 2018	CNKI		External use
Wang, C.C.; Chen, S.T.; Wu, S.J.; Chen, P.; Yen, C.J.; Wang, W.C.	Wang Wanchun's Experience in Comprehensive Treatment of Snake Bites	Guangming Journal of Chinese Medicine, 2017	CNKI		Not related to longterm effects of

	with Traditional Chinese Medicine (Chinese)			snakebite envenomation in the full text
Yi, W.; Ching, F.H.; Su, C.T.	Application of standardized Chinese medicine hoop in patients with bite by viper (Chinese)	Medical Equipment, 2017	CNKI	Nursing
Chang, T.S.	Observation on effect of traditional Chinese medicine snake wound fumigant lotion on swelling of limbs caused by snakebite (Chinese)	Chinese Journal of Clinical Rational Drug Use, 2017	CNKI	External use
Chiang, Y.F.	Effect of steam bath treatment on limb swelling after snakebite envenomation (Chinese)	Clinical Journal of Chinese Medicine, 2015	CNKI	External use
Jiang, H.Y.; Rong, C.L.; Pan, J.E.; Zhong, J.R.; Xie, W.Q.	Clinical observation of curative effect of the WAIXIYIFANG of traditional Chinese medicine combined with different leaf mixture in treating local swelling on the limbs caused by venomous snake bites (Chinese)	China Modern Medicine, 2013	CNKI	External use
Chuang, H.; Chen, T.F.; Ho, H.C.	Shejue Jiedu Powder as an adjuvant treatment for 47 cases of snake bite (Chinese)	Guangming Journal of Chinese Medicine, 2013	CNKI	External use
Yao, K.M.; Hu, J.H.	Therapeutic effect of blood purification combined with external application of traditional Chinese medicine on 33 cases of severe poisonous snake bites (Chinese)	Chinese Journal of Integrated Traditional and Western Nephrology, 2012	CNKI	External use
Wu, T.C.; Chou, W.C.; Liu, Y.P.; Yu, C.; Chiang, L.Y.	Practice of standard traditional Chinese herbs hooping care in patients with poisonous snakebite (Chinese)	Chinese Nursing Research, 2012	CNKI	Nursing
Huang, N.; Wu, K.C.; Yang, Y.L.; Su, Y.H.	Advances in Research Chinese Medical Treatment of Snakebite (Chinese)	Journal of Liaoning University of Traditional Chinese Medicine, 2010	CNKI	Not related to longterm effects of snakebite

					envenomation in the full text
Li, Y.L.	Treatment of 256 cases of poisonous snake bites with traditional Chinese medicine and homemade negative pressure drug inhaler (Chinese)	Journal of Emergency in Traditional Chinese Medicine, 2004	CNKI	No abstract available	
Wang, S.P.	Antivenoms combined with traditional Chinese medicine for 114 cases of snake bite (Chinese)	Journal of Snake, 2003	CNKI	No abstract available	
Li, H.L.	Clinical Observation on 30 Cases of Venomous Snake Bites Treated by Integrated Traditional Chinese and Western Medicine (Chinese)	Chinese Journal of Integrated Traditional and Western Medicine in Intensive and Critical Care, 1994	CNKI		External use
Yang, C.C.	Traditional Chinese medicine Semen Euphorbiae Lathyridis for snake bite (Chinese)	Henan Traditional Chinese Medicine, 1982	CNKI	Not available for full text	
Fang, Y.Y.; Li, H.L.	Traditional Chinese medicine Hsiung-Chih-San for snake bite (Chinese)	Jiangxi Medical Journal, 1965	CNKI	Not available for full text	
Shih, T.Y.; Chen, C.; Yu, Y.; Chu, M.S.	Effect of continuous moist therapy with Chinese medicine in treating snake bite (Chinese)	Journal of Traumatic Surgery, 2018	CNKI		External use
Cheng, P.Y.; Cheng, S.L.	Observation and Nursing of Traditional Chinese Medicine Steam Bath Formula in Treating Local Swelling and Pain Caused by Venom Snake Bites (Chinese)	Nei Mongol Journal of Traditional Chinese Medicine, 2015	CNKI		External use
Luan, K.T.; Tan, Y.A.; Li, C.K.	Treatment of Snakebite in Community Hospital (Chinese)	Journal of Snake, 2012	CNKI		External use

Li, H.; Hsiao, C.L.; Chou, C.; Sung, H.; Feng, C.L.	Effects of different medical care on efficacy of Chinese encircles spreads for local puncturing caused by snakebite (Chinese)	Nursing Practice and Research, 2010	CNKI		Nursing
Kang, N.	Analysis of Clinical Data of Integrated Traditional Chinese and Western Medicine in Treating Venomous Snake Bites (Chinese)	Chinese Journal of Ethnomedicine and Ethnopharmacy, 2009	CNKI		Not related to longterm effects of snakebite envenomation in the full text
Sung, H.; Chuan, L.; Liu, H.L.; Chou, C.; Hsiao, C.L.	Observation of curative effect of modified dressing on local tissue damage caused by bite (Chinese)	Journal of Emergency in Traditional Chinese Medicine, 2008	CNKI		External use
Cho, H.Y.; Kung, P.S.	Experience in rescue of 291 cases of poisonous snake bite by integrated traditional Chinese and western medicine (Chinese)	China Journal of Modern Medicine, 2005	CNKI		Nursing
Hsu, Y.H.	Traditional Chinese medicine as a main treatment in 35 cases of myocardial damage caused by snake bite (Chinese)	Chinese Journal of Integrative Medicine, 1990	CNKI	Not available for full text	
Wang, H.M.	Traditional Chinese medicine as a main treatment in 124 cases of snakebite envenomation (Chinese)	Journal of Anhui Traditional Chinese Medical College, 1993	CNKI	Not available for full text	
Li, C.W.	100 cases of snakebite envenomation treated by Chinese herbal medicine Lindernia crustacea (Chinese)	Guangxi Journal of Traditional Chinese Medicine, 1984	CNKI	Not available for full text	
Wang, P.P.	Effect of ear-buried beans on relieving pain in 35 patients with snake bite (Chinese)	Renowned Doctor, 2019	CNKI		No related to TCM
Lan, P.; Hung, H.P.; Chen, Y.; Lai, L.C.; Tu, W.	Effect analysis of different Traditional Chinese Medicine external methods in the	Zhejiang Journal of Traumatic Surgery, 2019	CNKI		External use

	treatment of patients with hemotoxic snakebite envenomation (Chinese)				
Hsieh, H.H.	Therapeutic effect of external application of traditional Chinese medicine on swelling and pain in limbs of snake bites in 38 cases (Chinese)	Hunan Journal of Traditional Chinese Medicine, 2013	CNKI		External use
Chen, S.; Yeh, H.T.; Hsiung, K.	Clinical Observation of Treatment Effect on Acute Respiratory Failure Patients by Traditional Chinese and Western Medicine (Chinese)	Journal of Snake, 2008	CNKI		Not related to longterm effects of snakebite envenomation in the full text
-	Chinese Medicine Information (Chinese)	Journal of Traditional Chinese Medicine, 1990	CNKI	No abstract available	
Chou, W.C.; Wu, T.C.; Liu, Y.P.; Yu, C.; Tang, L.	Nursing analysis of 22 cases with poisonous snake bite handling with Traditional Chinese herbs hooping (Chinese)	Chinese Nursing Research, 2010	CNKI		Nursing
Wang, W.	Report on 36 Cases of Detoxification and Detoxification of Traditional Chinese Medicine for Treatment of Snake Bites (Chinese)	Clinical Journal of Traditional Chinese Medicine, 2004	CNKI		Not related to longterm effects of snakebite envenomation in the full text
Tseng, S.Y.	18 cases of snakebite envenomation treated with traditional Chinese medicine (Chinese)	Journal of Traditional Chinese Medicine University of Hunan, 1996	CNKI		Not related to longterm effects of snakebite envenomation in the full text
Tai, W.T.	Experience in treating snake bites (Chinese)	Journal of Guiyang College of Traditional Chinese Medicine, 1989	CNKI	Not available for full text	

-	Rhizoma Paridis - King of Treatment of Snakebite envenomation (Chinese)	Chinese Medicine Modern Distance Education of China, 2013	CNKI	No abstract available	
Lu, T.K.; Liao, T.Y.; Teng, W.C.	Antivenom combined with traditional Chinese medicine for 114 cases of snake bite (Chinese)	Guangdong Medical Journal, 2002	CNKI		Use internal, injection and external TCM at the same time
Kao, T.	Clinical experience of steam bath with traditional Chinese medicine in treating joint stiffness at the late stage of fracture (Chinese)	Zhejiang Journal of Traumatic Surgery, 2012	CNKI		Not related to snakebite envenomation in the abstract
Shih, W.L.; Huang, H.P.; Wang, S.C.	Clinical study on TCM snakebite fumigating and washing lotion in treating limbs swelling after snakebite (Chinese)	Journal of Snake, 2012	CNKI		External use
Liu, M.C.	Sixteen flavors of traditional Chinese medicine for 25 cases of snake bite (Chinese)	Journal of Snake, 2001	CNKI		Acute satge/Use internal and external TCM at the same time
Yu, L.L.; Ho, C.C.	Clinical study of traditional Chinese medicine and Yao medicine in combination with regular western medicine on swelling and ulcer caused by snakebite (Chinese)	Pharmacy Today, 2016	CNKI		Not related to longterm effects of snakebite envenomation in the full text
Chang, L.F.; Tseng, C.Y.; Huang, P.; Chang, S.P.	Research Preparation and Clinic Application of Sheshang I Mixture (Chinese)	Journal of Snake, 2004	CNKI		External use
Fu, J.C.; Lin, C.C.	Experience in treating snake bites (Chinese)	Fujian Journal of Traditional Chinese Medicine (TCN), 1963	CNKI	Not available for full text	
Pan, L.P.; Wang, L.C.	Observation and nursing of traditional Chinese medicine medicated bath on skin ulcer of affected limb of snake bite (Chinese)	Modern Journal of Integrated Traditional Chinese and Western Medicine, 2008	CNKI		External use

Yang, H.; Huang, H.P.; Chiang, S.; Wang, C.C.	Clinical Drug Treatment of 87 Cases of Acute Renal Failure Caused by Snake Injury (Chinese)	Jilin Medical Journal, 2012	CNKI		Not related to longterm effects of snakebite envenomation in the full text
Ho, L.C.; Chen, H.	Introduce several Chinese herbs for treating snake wounds (Chinese)	Strait Pharmaceutical Journal, 2007	CNKI	Not available for full text	
Meng, H.	Mailuoning for the treatment of foot swollen by viper bite in 32 cases (Chinese)	Journal of Snake, 1999	CNKI		Not related to longterm effects of snakebite envenomation in the full text
Lu, L.M.; Lu, C.J.	Analysis of Detoxification of Lablab Semen Album. (Chinese)	Chinese Medicine Modern Distance Education of China, 2014	CNKI		Not related to snakebite envenomation
Ling, K.M.	Clinical observation of external application of Maotian Qi and Da Huang in the treatment of snake wounds (Chinese)	Modern Journal of Integrated Traditional Chinese and Western Medicine, 2009	CNKI		External use
Wu, F.; Wang, W.C.; Sun, P.; Chiang, H.; Liu, L.; Wang, H.	Experience of traditional Chinese medicine decomposing debridement in treating snake wound ulcers (Chinese)	Guangming Journal of Chinese Medicine, 2019	CNKI		External use
Chiang, H.P.	Treatment of 34 Cases of Critical Illness by Five-step Snake Bite with Integrated Traditional Chinese and Western Medicine (Chinese)	Journal of Snake, 1998	CNKI		Not related to longterm effects of snakebite envenomation in the full text
-	Latest Chinese Patent Abstracts (Chinese)	Science-Technical on Middle-Small Business, 2000	CNKI	Not available	

	for full text

TCM: Traditional Chinese Medicine; NDLTD: National Digital Library of Theses and Dissertations in Taiwan; CNKI: China National Knowledge Infrastructure.
